# Biological treatment of tannery wastewater by using salt-tolerant bacterial strains

**DOI:** 10.1186/1475-2859-7-15

**Published:** 2008-04-29

**Authors:** Senthilkumar Sivaprakasam, Surianarayanan Mahadevan, Sudharshan Sekar, Susheela Rajakumar

**Affiliations:** 1 Chemical Engineering Department, Central Leather Research Institute, Adyar, Chennai-20, India; 2 Microbiology Division, Central Leather Research Institute Adyar, Chennai-20, India

## Abstract

**Background:**

High salinity (1–10% w/v) of tannery wastewater makes it difficult to be treated by conventional biological treatment. Salt tolerant microbes can adapt to these saline conditions and degrade the organics in saline wastewater.

**Results:**

Four salt tolerant bacterial strains isolated from marine and tannery saline wastewater samples were identified as *Pseudomonas aeruginosa, Bacillus flexus, Exiguobacterium homiense *and *Staphylococcus aureus*. Growth factors of the identified strains were optimized. Tannery saline wastewater obtained from a Common Effluent Treatment Plant (CETP) near Chennai (southern India) was treated with pure and mixed consortia of four salt tolerant bacterial strains. Experiments with optimized conditions and varying salt content (between 2 and 10% (w/v) were conducted. Salt inhibition effects on COD removal rate were noted. Comparative analysis was made by treating the tannery saline wastewater with activated sludge obtained from CETP and with natural habitat microbes present in raw tannery saline wastewater.

**Conclusion:**

Salt tolerant bacterial mixed consortia showed appreciable biodegradation at all saline concentrations (2%, 4%, 6%, 8% and 10% w/v) with 80% COD reduction in particular at 8% salinity level the consortia could be used as suitable working cultures for tannery saline wastewater treatment.

## Background

Tannery saline wastewater, a primary effluent stream in leather processing industry is generated by soaking the salt-laden hides and skins in fresh water to remove excess salt. The presence of high salinity (1–10% NaCl by wt) in this waste stream hinders treatment by biological means [1]. A biomass sludge adapting to varying saline concentrations (salt tolerant strains) is required to degrade the dissolved organics present in tannery saline wastewater. Several groups have made significant contribution to biological treatment of saline wastewater [2-6]. Kargi and Dincer treated waste waters rich in halogenated organics at different salt concentrations and showed COD removal decreased with increasing salt concentration [7]. Woolard and Irvine studied the treatment of hypersaline wastewater by a moderate halophile in a sequencing batch reactor and in a biofilm reactor [8,9]. Up to 99% phenol removal was obtained from a 15% saline wastewater. Again Kargi and Dincer [10] employed *Zooglea ramigera *to study salt inhibition on COD removal in synthetic wastewater in an aerobic fed-batch reactor and further used a halophilic culture, *Halobacter halobium *to overcome the problem of plasmolysis and study the performance of biological treatment under high salt concentration [11].

Major problems encountered in biological treatment of saline wastewaters were (i) a limited extent of adaptation, as conventional cultures could not be effectively used to treat saline wastewaters of values higher than 3–5% (w/v) salt. (ii) salt adaptations of cultures were easily lost when subjected to salt-free medium. (iii) changes in ionic strength cell disruptions due to shifts in salt concentration, from 0.5 to 2% (w/v), caused significant reductions in system performance [12]. (Even with acclimatized cultures, satisfactory performance required a constant ionic composition). (iv) rapid changes in salt concentrations created adverse effects more than gradual changes. Normalization to constant salt concentration is essential before saline wastewaters were treated. Reduced degradation kinetics too occurred. Thus available information indicated that the removal of BOD by biological treatment processes is reduced at salt concentrations above 8,000 mg l^-1^. So saline wastewater should be treated at lower food to micro-organism (F/M) ratios, or higher than usual bacteria Mixed Liquor Volatile Suspended Solids (MLVSS) concentrations. Rapid changes in salt concentration cause adverse effects more than gradual changes. The treatment process should provide sufficient hydraulic retention time (HRT) to even out changes in salt concentration. In recent years there have been a few studies on biological treatment of tannery soak liquor. Lefebvre et al [13] made an attempt to treat tannery saline wastewater biologically. They studied the microbial diversity of hypersaline tannery wastewater [14] and anaerobic digestion of tannery soak liquor in an Upflow Anaerobic Sludge Blanket (UASB) reaching 78% COD removal [15]. Unidentified microbial consortia and halophillic/moderate halophillic cultures were used.

In our study, the focus was on salt concentration on biological treatment of commercial tannery saline wastewater inoculated with salt-tolerant bacterial strains and their mixed consortia isolated and identified from saline environments. Since the salinity levels of tannery saline wastewater varied everyday, a detailed study was undertaken over a broad range of changes in saline concentration. Corresponding COD removal by the identified salt tolerant strains was also followed. The results were compared with those obtained when commercial tannery activated sludge was treated. Variation in COD removal rate and its efficiency with salt concentration were determined. This was perhaps the first attempt on the biological treatment of tannery saline wastewater employing isolated and identified salt tolerant bacterial strains individually and in mixed consortia.

## Experimental

### Materials

#### Salt tolerant bacterial strains

Samples taken for salt tolerant bacterial isolation contained seawater, marine soil, salt lake water (15% and 20% NaCl w/v) and salt lake sediment clay; Samples were collected from salt lake around coastal areas in Mahabalipuram (60 km from Chennai) and from Adyar beach in Chennai. Tannery saline wastewater samples were collected from a commercial tannery at Chromepet, Chennai, India. Serial dilution technique was adopted and the separated colonies (> 10^-6 ^dilution) were spread plated in saline nutrient agar plates (varying salt concentrations from 0–25% NaCl (w/v), covering all salt tolerant, moderate and extreme halophilic ranges) and incubated at 37°C for 24 h. Screening of potent salt tolerant bacterial strains was done based on salt tolerance limit and time of adaptation. The isolated salt tolerant bacterial strains were identified by 16S rRNA analysis (MWG AGBiotech, Bangalore, India) (See additional files 1, 2, 3 and 4)

#### Culture medium

Isolation of salt tolerant bacterial colonies from saline samples was made with nutrient agar (Himedia, MO12) media with the following composition (in g l^-1^): Peptone; 10, NaCl; 5, Beef extract; 5 and Agar; 15. Solid NaCl was added to the agar to obtain the desired salt concentration and pH was adjusted to 7.0. Cultivation and optimization experiments were performed with nutrient broth (NB) media with the following composition: Peptic digest of animal 5 g l^-1^, Yeast extract 1.5 g l^-1^, Beef extract 1.5 g l^-1^, NaCl 5 g l^-1^. Dunda's media (for salt tolerant bacterial cultivation) composed of MgSO_4_. 7H_2_O (20 g l^-1^), NaCl (150 g l^-1^), Trisodium citrate dihydrate (3 g l^-1^) CaCl_2_. 2H_2_O (1 g l^-1^), Peptone (10 g l^-1^) and Yeast extract (1 g l^-1^) was also used in media optimization experiments.

#### Inoculum preparation

Frozen cultures of pure salt tolerant bacterial colonies stored in agar slants were inoculated on 5 ml nutrient broth media at aseptic conditions. The inoculated broth was incubated in an orbitary shaker (Scigenics, India), 150 rpm and 37°C, for 24 h. Well-grown culture suspensions with uniform concentration (absorbance at 600 nm ≈ 1) were used as sources of inoculum for all growth optimization and wastewater experiments.

#### Analytical methods

Salinity was measured by the argentometric method [16]. Total dissolved and suspended solids were determined according to the Standard Methods for Analysis of Water and Wastewater [17]. Protein content was determined by the Total Kjeldahl Nitrogen (TKN) method. Chemical Oxygen Demand (COD) and Biochemical Oxygen Demand (BOD) analyses on the clear supernatant (centrifuged at 6000 × g for 30 min) of samples were performed according to standard methods. Sufficient amount of HgSO_4 _(HgSO_4_/Cl = 10) was added to the samples to overcome the chloride ion interference in COD measurements. Biomass concentrations (for growth optimization studies) were determined by reading the absorbance of samples in a UV-spectrophotometer (Shimazdu, UV-2101 PC) at 600 nm.

#### Optimization experiments

Experiments were carried out in shaking flasks for optimization of several growth parameters (Temperature, pH, inoculum conc., salinity, and agitation rate) in synthetic nutrient media. Growth studies on nutrient media favoring optimal biomass growth were also performed. In all optimization studies, the samples (collected at regular time intervals) were analyzed for biomass growth by monitoring the absorbance in UV-spectrophotometer (Shimadzu, UV-2101PC) at 600 nm. Growth curves were drawn (plotting absorbance against time) for each growth condition and the optimum value of each parameter was fixed by comparing the growth curves for higher biomass yield. The results of growth optimization studies for the identified strains are given in Table 1.

**Table 1:** Optimized growth parameter results of the halotolerant bacterial isolates

**Halotolerant bacterial strains**	**Media**	**Salinity (w/v)**	**Inoculum Conc. (v/v)**	**pH**	**Temperature (°C)**	**Agitation rate (rpm)**
*P.aeruginosa*	NB	5%	4%	7.5	37°C	160
*B.flexus*	NB	15%	4%	8	32°C	160
*E.homiense*	NB	10%	2%	7.5	32°C	160
*S. aureus*	NB	10%	2%	7	32°C	160

#### Wastewater

Tannery saline wastewater samples were obtained from the collection tank of a Common Effluent Treatment Plant (CETP, Pallavaram, Chennai) designed to treat wastewater generated from nearby tanneries. The wastewater was characterized in terms of: total soluble and suspended solids (TDS & TSS), COD, BOD, salinity, TKN and pH respectively (see "Analytical methods"). (See Table 2 for composition).

**Table 2:** Characteristics of raw tannery saline wastewater

**Parameter**	**Composition**
COD (ppm)	2512
BOD (ppm)	1484
TSS (mg l^-1^)	7258.2
TDS (mg l^-1^)	30971.7
Chloride (mg l^-1^)	16559.2
TKN (mg l^-1^)	1200
pH	7.45

#### Biological tannery saline wastewater treatment

The treatment of tannery saline wastewater was performed in shaking flasks (100 ml batch size) at varying NaCl concentrations viz., 2%, 4%, 6%, 8% and 10% (w/v). The non-sterilized tannery saline wastewater samples were taken separately and the efficiency of COD removal by isolated salt tolerant bacterial strains was studied by inoculating them as pure monocultures. Samples were also run with mixed isolated salt tolerant bacterial consortia and tannery activated sludge. Blank runs were made with natural organisms present in raw tannery saline wastewater in similar growth conditions. For each run, 2% (v/v) of cell suspension was used to inoculate 100 ml tannery saline wastewater to which were added varying NaCl concentrations in 200 ml Erlenmeyer flasks. This was then incubated at 37°C and 150 rpm in an orbitary shaker. Orbitary motion of the samples sustained sufficient amount of oxygen supply (Dissolved Oxygen > 2 mg/l) to ensure aerobic conditions throughout the experiment. Samples were taken out daily and centrifuged at 10000 × g for 10 min in a Sigma 3 MK refrigerated centrifuge and the supernatant was taken up for COD measurements.

## Results and discussion

Biochemical analysis and 16s rRNA analysis of the isolated salt tolerant bacterial colonies helped to identify them as *Pseudomonas aeruginosa *(isolate from tannery saline wastewater), *Bacillus flexus *(isolate from marine soil), *Exiguobacterium homiense *(isolate from salt-lake saline liquor) and *Staphylococcus aureus *(isolate from seawater). Occurrence of *Pseudomonas aeruginosa *in marine sources and its salt tolerance property had already been reported [18].

### Growth optimization

Optimization experiments were performed for the growth of halotolerant bacterial isolates at shaker level to determine the growth factors simulating higher biomass yield. Composition of media, salinity, inoculum concentration, pH, temperature, and agitation rate were the parameters optimized. Samples were collected at regular time intervals; the biomass growth was monitored by recording the absorbance at 600 nm in spectrophotometer. The absorbance values were plotted against time to obtain growth curves.

Values showing an exponential phase and high biomass yield were found to be optimum. The isolated strains (*P.aeruginosa, B.flexus, E.homiense and S.aureus*) were grown in two different media (NB & Dundas) under similar physical environment and growth was ascertained by measuring optical density (at 600 nm) of culture for 24 h (results not shown). It was observed that bacterial growth was maximum in nutrient broth- it being an indication that the halotolerant bacterial strains were proteolytic in nature, readily degrading the large amount of peptone, present in both media. Also, the halotolerant bacterial strains exhibited a strong stationary phase up to 8 h in NB media as compared to the observed phase in Dunda's medium. This pointed to the long survival ability of the cultures; NB was also found to be the ideal medium. Salinity optimization was a parameter of greater importance as the halotolerant bacterial strains were to be employed for biodegradation of tannery saline wastewater.

Salinity studies showed that *P.aeruginosa *as well as *S.aureus *adapted up to 5% (w/v) and *B.flexus, E.homiense *over a wide range (5%, 10% & 15%) of salinity (results not shown). This meant that all the identified strains (*P.aeruginosa*, *B.flexus, E.homiense *and *S.aureus*) were salt tolerant. Inoculum concentrations were changed from 2 – 10% (v/v) for each strain, keeping other growth parameters constant. Samples were withdrawn at regular time intervals and analyzed for growth by recording absorbance value at 600 nm (results not shown). Maximum biomass yield was taken as criterion to find the optimum value. A 4% (v/v) inoculum conc. was found to be optimum for *P.aeruginosa *and *B.flexus *and 2% for *E.homiense *and *S.aureus*. Biological saline wastewater treatment showed improved results when operated at low F/M for high salt concentrations [19]. Temperature optimization studies proved that all the 4 halotolerant bacterial isolates were found to be mesophillic (results not shown) as enhanced biomass production was observed in this temperature range (32–37°C). As most of effluent treatment plants were operated in ambient conditions (especially in tropical countries like India were normal day temperature was ≥ 30°C), the isolates could actively degrade the tannery saline wastewater. pH optimization results proved that all the halotolerant bacterial isolates were found to be nuetrophile (pH 7.0 – 8.0). In soaking operation, common salt was the main ingredient and it imparted a neutral pH to tannery wastewater. Hence no pH correction was needed when these strains were employed for biodegradation of tannery saline wastewater (they easily adapted). Optimization of agitation rate for the growth of identified strains was taken up at different values viz., 100, 120, 140, 160 and 180 rpm. This small increment in agitation rate showed significant variation in growth rate of respective strains. All the four salt tolerant strains showed optimal growth at 160 rpm and a further increase in agitation rate caused observable fall in growth profile (results not shown). This could be attributed to the effect of shear rate on cell wall, resulting in cell damage. Optimization results indicated that the identified halotolerant bacterial strains could readily degrade tannery saline wastewater. The summaries of experiments for the 4 halotolerant bacterial strains are in Table 1.

### Tannery saline wastewater treatment

**Figure 1:**
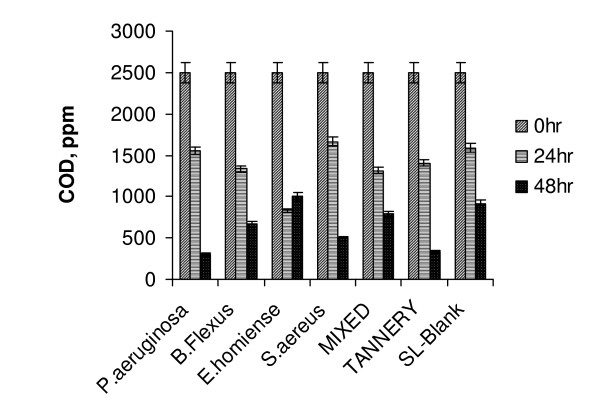
Variation in COD reduction of tannery saline wastewater (2% Salinity, T = 37°C, pH 7.5, 200 rpm).

Tannery saline wastewater degradation studies were done as batch experiments (incubated shaking flask) with pure monocultures of *P.aeruginosa, B.flexus, E.homiense and S.aureus *and their mixed consortia. In CETPs, tannery saline wastewater was segregated generally separately and treated in solar ponds to prevent degeneration of tannery sludge in the bioreactor. In order to analyze the efficiency of the identified strains reported on treatment of tannery saline wastewater, comparative studies were performed employing activated tannery sludge and natural habitat microbes (those present in the raw effluent) separately. Batch experiments on tannery saline wastewater degradation were performed at optimized growth conditions of respective strains as given in Table 1 (inoculum dosage (2% v/v), pH of 7.0, 37°C and at 200 rpm). The presence of 1.7% of NaCl (by wt) showed that (table 2) tannery saline wastewater was slightly saline. High TKN and BOD values further indicated the suitability of the collected sample for biodegradability studies. In batch experiments, salt concentration was changed from 2–10% (w/v) to minimize commercial tannery saline wastewater salt composition. Experimental runs were continued till appreciable COD removal (> 80%) was obtained. Samples were taken at regular time intervals, analyzed for COD value and chloride effects were masked by addition of HgSO_4 _in proportion to chloride concentration. Each set of experiments was repeated for three times and only mean values of the results were discussed. Statistical analysis (ORIGIN software) of results for replicas showed a minimum standard deviation range of ± 3% for most of the experiments. (Fig. 1 depicts the variation in COD reduction of tannery saline wastewater salinity, 2% w/v) by salt tolerant bacterial isolates, mixed salt tolerant consortia and tannery sludge). A maximum degradation of 87.6% was observed for *P.aeruginosa *compared to tannery sludge (68.5%). Studies on effect of salinity already showed that *P. aeruginosa *was effective in adapting to low salt concentrations (> 5% w/v) compared with the other strains reported. This finding was also reflected in the experimental results obtained from biodegradation of tannery saline wastewater and confirmed that *P.aeruginosa was *a suitable working strain for treatment of tannery effluent of low saline concentration.

**Figure 2:**
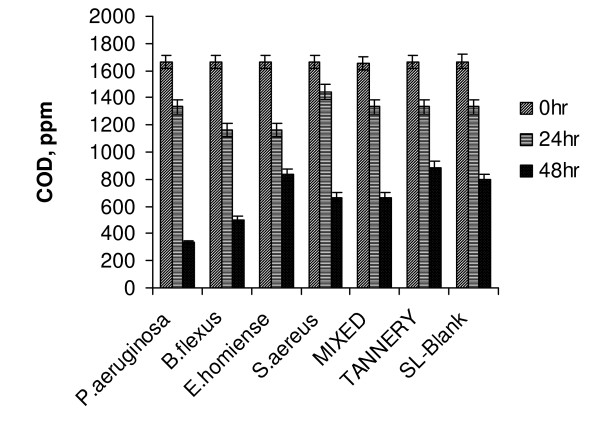
Variation in COD reduction of tannery saline wastewater (4% Salinity, T = 37°C, pH 7.5, 200 rpm).

**Figure 3:**
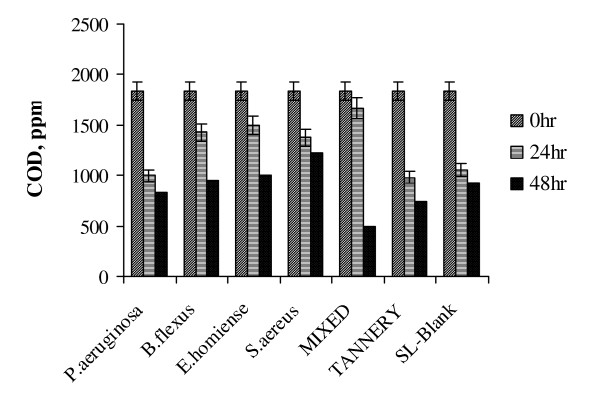
Variation in COD reduction of tannery saline wastewater (6% Salinity, T = 37°C, pH 7.5, 200 rpm).

The results obtained for treatment of tannery saline wastewater at 4% (w/v) saline condition also showed *P. aeruginosa *to be an ideal strain. A higher COD removal (of 80%) was noted as compared with other strains and mixed culture. At 4% salinity level, although it took two days for *P.aeruginosa *to values with adapt as well as achieve 80% degradability, it turned out to be an effective strain (Fig. 2). Mixed salt tolerant consortia showed 59.8% COD removal at 4% (w/v) saline concentration. In both 2% and 4% saline concentrations, mixed consortia did not show appreciable degradation of tannery effluent. As the mixed consortia had all the four identified halotolerant strains, the inhibition in growth/enzyme secretion due to competition for earlier adaptation on saline wastewater could have been dominant at low saline concentrations This 'competition' suppressed the activity of the respective strains resulting in poor performance in low salt regime. (Fig. 3 shows the comparative profile of COD removal in tannery saline wastewater by identified strains and mixed consortia at 6% (w/v) saline concentration). It was observed that mixed salt tolerant bacterial consortia effectively degraded tannery saline wastewater with 6% (w/v) salinity level (83% degradation).

**Figure 4:**
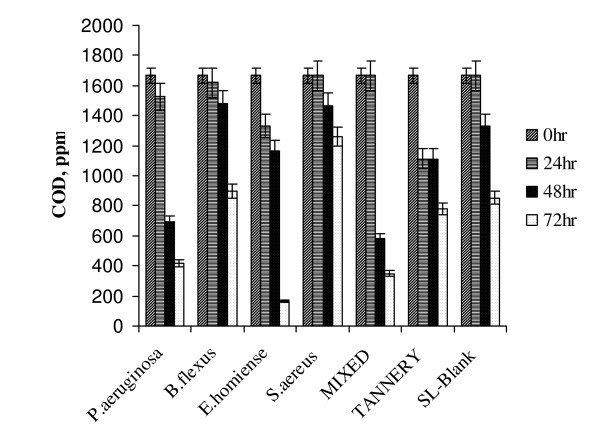
Tannery Saline wastewater degradation studies on shaking flasks for 3 days duration (8% Salinity, T = 37°C, pH 7.5, 200 rpm).

The above data suggest that cumulative salt tolerance activity of identified isolates in the consortia was pronounced compared with individual activity of respective strains. This finding further proved that mixed consortia could be applied effectively for treatment and management of moderate strength tannery saline waste stream. (A comparative COD removal profile for treatment of tannery saline effluent at 8% (w/v) is given in Fig. 4). Analysis of results showed that *E.homiense *exhibited a higher COD removal (90%). Mixed salt tolerant consortia also showed an appreciable COD removal (80%) at 8% (w/v) salinity concentration, but not as efficiently as with *E.homiense*. The individual efficiency of *E.homiense *to degrade tannery saline wastewater could have been inhibited when it was present in mixed consortia, due to substrate competition and initial biomass density.

**Figure 5:**
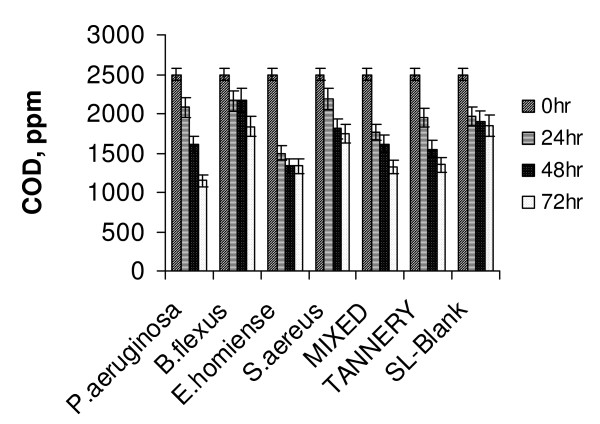
COD reduction of tannery saline wastewater in shaking flasks for 3 days (10% Salinity, T = 37°C, pH 7.5, 200 rpm).

**Figure 6:**
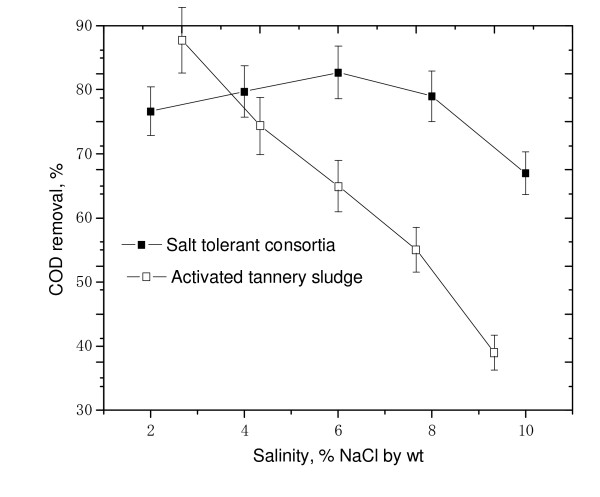
Comparative profiles of % COD removal of tannery soak liquor by identified salt tolerant bacterial consortia and tannery sludge.

A sudden fall in COD reduction was observed for all the strains (> 50%) when salinity level increased to 10% (Fig. 5). This indicated that even a smaller increment in salinity (8 to 10%) could result in adverse effect on treatment efficiency. From the results given in Fig. 5, it could be observed that both *E.homiense *and mixed consortia exhibited similar COD removal (48%) at 10% salinity concentration. The experimental results of COD removal at 8% and 10% saline concentration further suggested that *E.homiense *employed in this study could be a moderately halophillic strain. In CETPs, average salt concentration of tannery wastewater had been found to vary between 2% and 6% and this covered salt tolerant regime only. The salt concentration hardly exceeded 8% (w/v) for effluent released from beam house operations of leather processing units and the identified mixed consortia reported in this study could be advantageously employed for efficient biological treatment of the saline waste stream. It could be observed from Fig(s) 1, 2, 3, 4, 5, that the treatment of tannery saline wastewater by natural habitat strains (blank) was not a feasible option. Efficiency of COD removal was observed to be less than 50% for different saline concentrations viz., 2%, 4%, 6%, 8% and 10% (w/v). From Fig(s) 2, 3, 4, 5, it could be observed that the conventional activated tannery sludge showed a low COD removal compared to the salt tolerant consortia. (Fig. 6 describes the comparative profile of % COD removal by activated sludge (tannery) and salt tolerant consortia). From Fig. 6, it could be observed that as the salinity increased from 2 to 4% (w/v), tannery sludge efficiency reduced to 60% from 80%; it reduced further to 45% for 6% (w/v) saline value. The COD removal results for tannery saline waste stream by natural habitat strains as well as activated tannery sludge proved they were not suitable and that specialized consortia (salt tolerant) were needed for efficient treatment. Our experimental findings supported the view that the identified salt tolerant bacterial consortia be considered as a suitable working culture for efficient biodegradation of tannery saline wastewater.

## Conclusion

Salt tolerant bacterial strains (*P.aeruginosa, B.flexus, E.homiense and S.aureus*) were isolated and identified from marine sources and tannery saline wastewater. Growth factors (media, inoculum conc., salinity, pH, temperature and agitation rate) were optimized for identified strains in batch experiments. Commercial tannery saline wastewater was characterized and biologically treated with both mono and mixed halotolerant bacterial isolates. Comparative analysis was done by degrading the tannery saline wastewater with active biomass sludge obtained from tannery and with natural habitat microbes present in raw tannery saline wastewater. The salt concentrations were varied from 2–10% (w/v) and a good biodegradability of tannery saline wastewater was observed with salt tolerant bacterial mixed consortia (80% COD reduction in 8% salinity). Increase in salt concentration to 10% resulted in deterioration of the treatment process and poor COD reduction (60% for mixed consortia). This study suggested the possibility of successful application of identified salt tolerant bacterial consortia for efficient degradation of tannery saline wastewater. Such application would compare well with other conventional biological treatment processes being used with activated sludge as a working culture.

## Authors' contributions

SS carried out the tannery wastewater biodegradation studies and drafted the manuscript. MS guided this work, analyzed the results and made technical correction of the manuscript. SS carried out the bacterial isolation, biochemical assays and COD estimation. SR participated in the design of experiments. All authors read and approved the final manuscript.

## Supplementary Material

Additional file 1 16S rRNA sequence of *P. aeruginosa*. The data provided represents the 16S rRNA sequence to identify the name of the bacterial strain. Click here for file

Additional file 2 16S rRNA sequence of *B. flexus*. The data provided represents the 16S rRNA sequence to identify the name of the bacterial strain. Click here for file

Additional file 3 16S rRNA sequence of *E. homienese*. The data provided represents the 16S rRNA sequence to identify the name of the bacterial strain. Click here for file

Additional file 4 16S rRNA sequence of *S. aureus*. The data provided represents the 16S rRNA sequence to identify the name of the bacterial strain. Click here for file
