# Supplementary figures and images for: Biological treatment of tannery wastewater by using salt-tolerant bacterial strains
Source: Microb Cell Fact. 2008 Apr 29;7:15. doi: 10.1186/1475-2859-7-15 (PMC2397378; doi:10.1186/1475-2859-7-15)

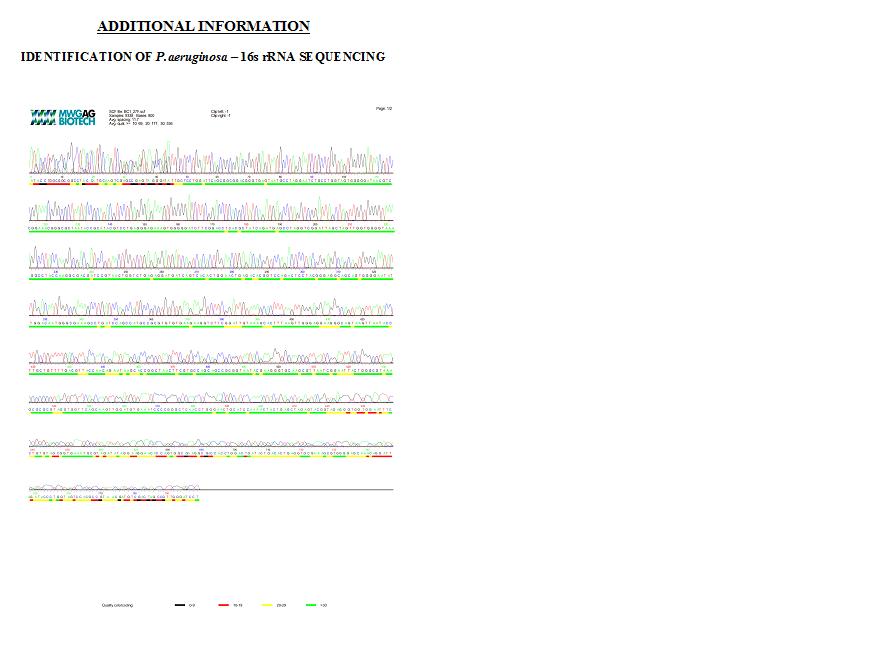

Supplement: Additional file 1 — 16S rRNA sequence of P. aeruginosa. The data provided represents the 16S rRNA sequence to identify the name of the bacterial strain. [file 1475-2859-7-15-S1.jpeg]

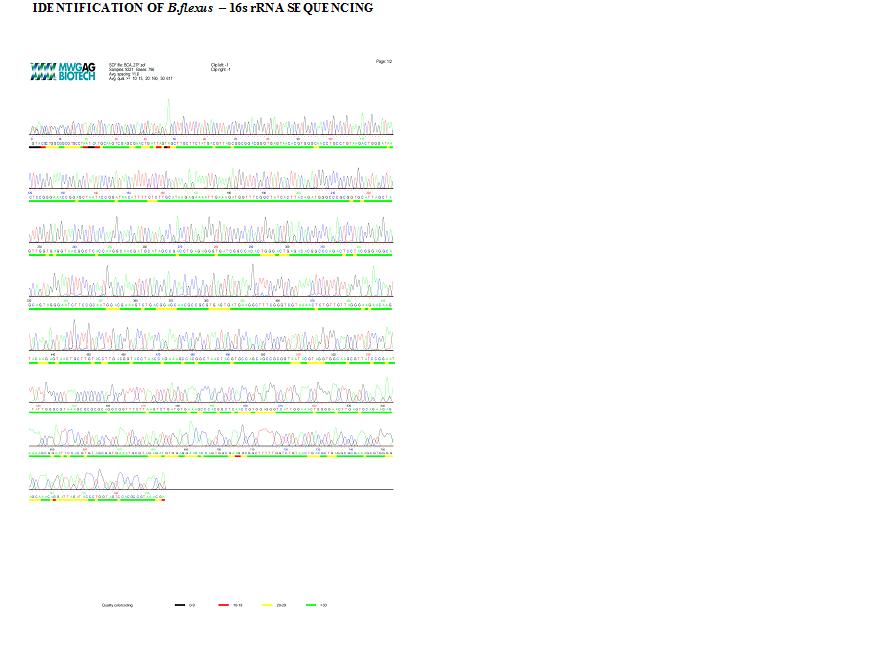

Supplement: Additional file 2 — 16S rRNA sequence of B. flexus. The data provided represents the 16S rRNA sequence to identify the name of the bacterial strain. [file 1475-2859-7-15-S2.jpeg]

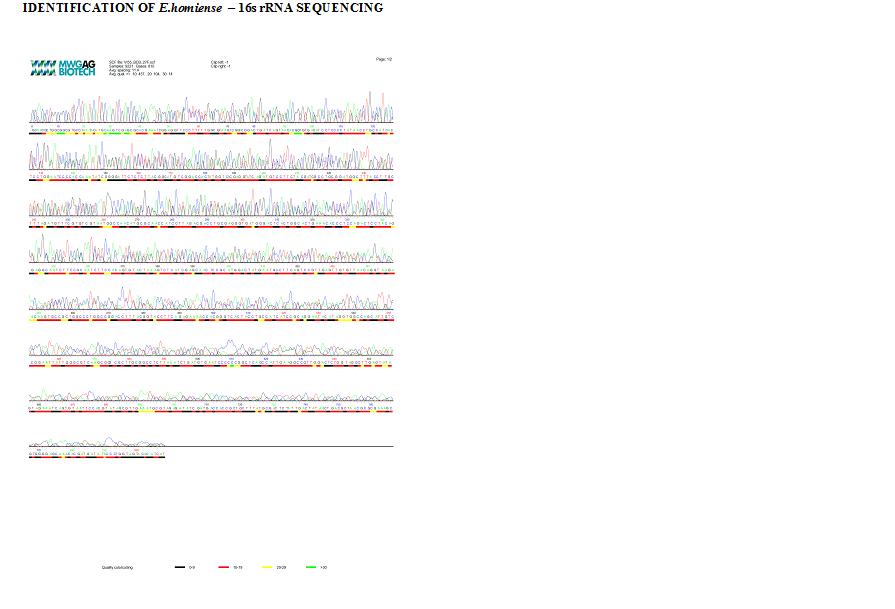

Supplement: Additional file 3 — 16S rRNA sequence of E. homienese. The data provided represents the 16S rRNA sequence to identify the name of the bacterial strain. [file 1475-2859-7-15-S3.jpeg]
