# Supplementary material for: Biological treatment of tannery wastewater by using salt-tolerant bacterial strains
Source: Microb Cell Fact. 2008 Apr 29;7:15. doi: 10.1186/1475-2859-7-15 (PMC2397378; doi:10.1186/1475-2859-7-15)
Supplement: Additional file 4 — 16S rRNA sequence of S. aureus. The data provided represents the 16S rRNA sequence to identify the name of the bacterial strain. [file 1475-2859-7-15-S4.doc]

***Staphylococcus aureus* - Identification**

**Service:**  Bacterial Identification Service based on 16S rDNA data

**Steps Involved: 1.** Genomic DNA was isolated from the pure culture pellet provided by the scientist.

**2.** Using consensus primers, the ~1.5 kb 16S rDNA fragment was amplified using high-fidelity PCR Polymerase.

**3.** The PCR product was bi-directionally sequenced using the forward, reverse and an internal primer.

**4.** Sequence data was aligned and analyzed for finding the closest homologs for the microbe.

**Result:**

1. Based on nucleotides homology and phylogenetic analysis the Microbe (Sample: **CLRI**) was detected to be **(*Staphylococcus aureus)*,** GenBank Accession Number: **AB219154.**
2. Nearest homolog species was found to be ***Staphylococcus pulvereri* (**Accession No. **AB009942).**
3. Information about other close homologs for the microbe can be found from the Alignment View table.

**ALIGNED SEQUENCE DATA:** **(1485 bp)**

GATGAACGCTGGCGGCGTGCCTAATACATGCAAGTCGAGCGAACAGATGAGAAGCTTGCTTCTCTGATGTTAGCGGCGGACGGGTGAGTAACACGTGGGTAACCTACCTATAAGACTGGGATAACTCCGGGAAACCGGGGCTAATACCGGATAATATATTGAACCGCATGGTTCAATGTTGAAAGACGGTTTCGGCTGTCACTTATAGATGGACCCGCGCCGTATTAGCTAGTTGGTAAGGTAACGGCTTACCAAGGCAACGATACGTAGCCGACCTGAGAGGGTGATCGGCCACACTGGAACTGAGACACGGTCCAGACTCCTACGGGAGGCAGCAGTAGGGAATCTTCCGCAATGGGCGAAAGCCTGACGGAGCAACGCCGCGTGAGTGACGCACGCCTTAGGATCGTAAAACTCTGTTGTTAGGGAAGAACAAATTTGTTAGTAACTGAACAAGTCTTGACGGTACCTAACAAAAAAGCCCCGGCTAACTACGTGCCAGCAGCCGCGGTAATACGTAGGTGGCAAGCGTTATCCGGAATTATTGGGCGTAAAGCGCGCGTAGGCGGTTTCTTAAGTCTGATGTGAAAGCCCACGGCTCAACCGTGGAGGGTCATTGGAAACTGGGGAACTTGAGTGCAGAAGAGGAGAGTGGAATTCCATGTGTAGCGGTGAAATGCGCAGAGATATGGAGGAACACCAGTGGCGAAGGCGGCTCTCTGGTCTGTAACTGACGCTGATGTGCGAAAGCGTGGGGATCAAACAGGATTAGATACCCTGGTAGTCCACGCCGTAAACGATGAGTGCTAAGTGTTAGGGGGTTTCCGCCCCTTAGTGCTGCAGCTAACGCATTAAGCACTCCGCCTGGGGAGTACGACCGCAAGGTTGAAACTCAAAGGAATTGACGGGGACCCGCACAAGCGGTGGAGCATGTGGTTTAATTCGAAGCAACGCGAAGAACCTTACCAAATCTTGACATCCTTTGACCGCTCTAGAGATAGAGTTTTCCCCTTCGGGGGACAAAGTGACAGGTGGTGCATGGTTGTCGTCAGCTCGTGTCGTGAGATGTTGGGTTAAGTCCCGCAACGAGCGCAACCCTTAAGCTTAGTTGCCATCATTAAGTTGGGCACTCTAGGTTGACTGCCGGTGACAAACCGGAGGAAGGTGGGGATGACGTCAAATCATCATGCCCCTTATGATTTGGGCTACACACGTGCTACAATGGATAATACAAAGGGCAGCGAACCCGCGAGGTCAAGCAAATCCCATAAAATTATTCTCAGTTCGGATTGTAGTCTGCAACTCGACTACATGAAGCTGGAATCGCTAGTAATCGTAGATCAGCATGCTACGGTGAATACGTTCCCGGGTCTTGTACACACCGCCCGTCACACCACGAGAGTTTGTAACACCCGAAGCCGGTGGAGTAACCTTTTATTAGGAGCTAGCCGTCGAAGGTGGGACAGATGATTGGGGTGAAGTCGT

**Percentage Homology based on Nucleotide Sequence:**

| **SL. No.** | **ISOLATES** | PERCENTAGE HOMOLGY | | | | | | | | | | |
| --- | --- | --- | --- | --- | --- | --- | --- | --- | --- | --- | --- | --- |
|  |  | **1** | **2** | **3** | **4** | **5** | **6** | **7** | **8** | **9** | **10** | **11** |
| **1** | CLRI | ***** | 99 | 99 | 98 | 98 | 98 | 98 | 97 | 96 | 99 | 98 |
| **2** | **AB219154** |  | ***** | 100 | 99 | 98 | 98 | 98 | 98 | 96 | 99 | 99 |
| **3** | **D83370** |  |  | ***** | 99 | 98 | 98 | 98 | 98 | 96 | 99 | 99 |
| **4** | **AB009942** |  |  |  | ***** | 98 | 98 | 98 | 98 | 96 | 99 | 99 |
| **5** | **AM062696** |  |  |  |  | ***** | 99 | 98 | 99 | 97 | 98 | 98 |
| **6** | **AJ421446** |  |  |  |  |  | ***** | 99 | 99 | 97 | 98 | 98 |
| **7** | **AB116135** |  |  |  |  |  |  | ***** | 99 | 97 | 98 | 98 |
| **8** | **AB212276** |  |  |  |  |  |  |  | ***** | 96 | 98 | 97 |
| **9** | AJ717376 |  |  |  |  |  |  |  |  | ***** | 96 | 96 |
| **10** | AY395014 |  |  |  |  |  |  |  |  |  | ***** | 98 |
| **11** | **AM062694** |  |  |  |  |  |  |  |  |  |  | ***** |

**PHYLOGENETIC TREE:**

**CLRI**

**AB219154 (*Staphylococcus aureus* st.:SSH39**)

**D83370 (*S. aureus*)**

**99**

**AB009942 (*Staphylococcus pulvereri* )**

**AM062696 (*Staphylococcus sciuri*)**

**AJ421446 (*S. sciuri*)**

**AB116135 (*S. sciuri* st.:T131)**

**100**

**99**

**AB212276 (*S. sciuri* )**

**AJ717376 (*Staphylococcus pasteuri*)**

**AY395014 (*S. lentus*)**

**AM062694 (*Staphylococcus vitulinus*)**

**100**

**100**

**100**

**100**

**72**
